# Primary care in Switzerland: evolution of physicians’ profile and activities in twenty years (1993–2012)

**DOI:** 10.1186/s12875-015-0321-y

**Published:** 2015-08-21

**Authors:** Christine Cohidon, Jacques Cornuz, Nicolas Senn

**Affiliations:** Department of Ambulatory Care and Community Medicine, University of Lausanne, Lausanne, Switzerland

## Abstract

**Background:**

According to the Organization for Economic Cooperation and Development, the Swiss healthcare system is one of the most effective in the world. Yet, as other occidental countries, it has to face the increase of chronic diseases frequency and its resulting cost, particularly for primary care (PC). However very few consistent data are available to describe PC features and its evolution over time. The aim of this study is to describe the evolution of the Swiss PC physicians’ (PCPs) profile and activities between 1993 and 2012.

**Methods:**

The date come from two independent European surveys carried out in Switzerland respectively in 1993 and 2012. Both surveys were cross-sectional ones and based on representative samples of 200 PCPs, interviewed by questionnaire.

**Results:**

In 20 years, PCPs became older (median age 46 *vs *56, *p* < 0.001) and more feminized (7 % *vs* 22 %, *p* < 0.001). Nowadays, they more often work in group practices (28 % *vs* 52 % in 2012, *p* < 0.001) and are more involved in other paid activities (28 % *vs *66 % in 2012, *p* < 0.001). All the PCPs have a computer in 2012 (78 % in 1993, *p* < 0.001) and it is mostly used for keeping records of consultations (47 %). The number of daily face-to-face contacts with patients decreased from 31 to 24 but the average length rose from 15 to 20 min (*p* < 0.001). PCPs provide fewer pediatric and gynecological services but their activity remains globally unchanged in other domains. The frequency of meetings with other disciplines decreased significantly (e.g. once/month face-to-face meets with ambulatory specialists: 78 % *vs *23 % in 2012, *p* < 0.001). The involvement of PCPs in follow-up and treatment of chronic disease globally little differed. In 2012, 8.5 % of the PCPs never performed any chirurgical acts (*vs* 0 % in 1993, *p* < 0.001).

**Conclusion:**

This study showed a substantial evolution of Swiss PC over the last twenty years in terms of socio-demographic, organizational and service provided. The main changes include: feminization and ageing, lower diversity in services provided, fewer but longer consultations. These changes may have important implications for patients’ management and will need to be considered for health planning purposes.

## Background

According to the Organisation for Economic Cooperation and Development, OECD, the Swiss healthcare system is one of the most effective and high-quality in the world [1]. Today, its main assets are related to care access, high quality in both hospital and ambulatory delivered care and large coverage by compulsory health insurance. This situation combined with the high economic level of the country allows Switzerland to offer one of the highest life expectancy in the world. Beside this idyllic situation, the downside is that the Swiss health system is also one of the most expensive in the world. Moreover very few data are available to describe primary health care features, its functioning and evolution over time in particular for primary care physicians (PCPs). For instance, no reliable national dataset exists to describe demographic characteristics of PCPs. Finally data enabling the description of their activities are really sparse [2, 3].

The Swiss ambulatory health care system is based on fee-for-service, mainly provided by independent private practitioners and outpatient services in hospitals. But the healthcare organization and functioning has evolved during these last decades. The two major changes occurred respectively in 1996 with the implementation of the Federal law on health insurance, LaMal [4], which stipulates a mandatory health insurance for all residents in Switzerland and in 2004 with the introduction of Tarmed, which standardizes medical fees throughout the country [5, 6]. Another element can be added: the implementation of a moratorium about the physicians’ installation between 2002 and 2011. During this period, it was not possible for the physicians working in the ambulatory sector to open a private practice, except if the needs were proven.

Nowadays, the Swiss health care organization system, as many industrialized countries, has to face up economic challenges. More and more expensive costs are generated because of the growing medical technicality and the ageing population leading to a higher burden of chronic diseases to support. In such situation, PCPs are in first line. In parallel, PCPs demography also evolves. Due to sociological changes, PCPs are generally more often women, older and less implanted in rural areas. The feminization of PC’s profession, with more part-time work combined with the reduction of trained PCPs is believed to lead to a PCPs shortage in Switzerland with the next years [7, 8]. Thus, the Swiss health care system has to adapt its functioning to the evolution in health needs with the constraints of limiting raising costs [9, 10]. To success in this challenge, it is necessary to improve the understanding of the PC system. In 1993 and 2012, Switzerland was involved in two Europeans surveys whom objective was to describe PC in all Europeans countries. This present study aims to use these data to describe the evolution of the Swiss PCPs’ profiles and activities in twenty years, between 1993 and 2012.

## Methods

### Study population

Data stem from two independent cross-sectional European surveys based on similar sampling methods and questionnaires. These surveys were coordinated by the same investigators at the Nivel Institute from Netherlands and carried out in Switzerland, respectively, in 1993 by the University of Saint-Gall and in 2012 by the University of Lausanne. The studies obtained the approval of the Swiss ethical review board.

#### The European study of tasks profiles of general practitioner (1993)

The 1993 data were collected through the Swiss participation in the European study “Task Profiles of General Practitioners” [11]. This study aimed to describe the range of services offered by general practitioners in European countries and their relationship to health care systems [12]. The project was funded by the European commission and involved 7233 responding physicians in thirty countries in Europe. The drawing of the sample and the organization of data collection through paper questionnaires was carried out at national level. The Swiss sample was drawn by a random sampling procedure from a national database of PCPs (the Swiss Medical Association, FMH, which covers around 95 % of the Swiss physicians), stratified by urban/rural area. A response rate of 50 % enabled to obtain a sample size of 200 Swiss PCPs. The representativeness of the sample was assessed comparing with national data on age and gender and was considered as good [13].

#### The quality and costs of primary care in Europe study (QUALICOPC, 2012)

The 2012 data were collected through the Swiss participation in the QUALICOPC study. This project aimed to analyze and compare how primary health care systems in 34 countries perform in terms of quality, costs and equity.

Surveys were held among PCPs in 31 European countries (EU 27 – except for France-, FYR Macedonia, Iceland, Norway, Switzerland, and Turkey) and 3 non-European countries (Australia, Canada, and New Zealand). In each country, a random nationally representative sample of around 220 physicians was drawn. Only one physician per practice or health centre was eligible to participate. In Switzerland, the participating physicians stemmed from a random sample of PCPs drawn from the two physicians’ associations in first care medicine (general medicine and general internal medicine) and stratified by canton, the SPAM network (response rate of 10 %) [14]. The representativeness in terms of gender, rural/urban implantation and age was cross-checked against national statistics and considered as satisfactory. Ethical approval was acquired in accordance with the legal requirements in each country. Details about the study protocol and questionnaire development have been published elsewhere [15, 16]. Data collection took place between January and June 2012 in Switzerland.

#### Data

In both surveys, PCPs were interviewed by self-administrated questionnaire sent by mail. The questionnaires were translated from the initial English master version by translators in the three national languages of Switzerland: German, French and Italian language.

For the present study, we only selected twenty-two questions that were strictly the same in both surveys (both formulations of questions and answers). These questions were related to two domains of health care indicators, according to Donabedian’s classification: structure indicators (“all the factors that affect the context in which care is delivered”) and process indicators (“all actions that make up healthcare”) [17]. Structure indicators were explored through socio-demographic features of PCPs in terms of sex, age, rural/urban practices location and language areas of Switzerland (German, French or Italian). The practices location was described by the PCP using five items: big city, suburbs, small town, semi-rural and rural area. The items were secondarily grouped in two categories: urban area (big city, suburbs, small town) and rural area (semi-rural and rural area). The age variable was dichotomized at the median value in the global sample. Questions related to output indicators were arranged in different areas:

#### General features

General practice characteristics were described through the following items: solo or group practice, unique activity as PCP or involvement in other paid activity (such as physicians in nursing homes, private companies, teaching activities…), salaried or self-employed activity and weekly workload as PCP (number of hours). The workload distinguished regular hours and after-hours (weekend days and nights).

The equipment and aims of use of computers and the regular meets with others health care professionals (kind of professionals and frequency) were explored.

#### Primary health care access

Care access was explored using different indicators: daily face-to-face patient contacts (number and duration), number of daily patient phone contacts with patients and weekly home visits. Finally, distance to other PCPs, outpatient clinics and hospital in terms of km was assessed (declared by PCPs).

#### Care continuity – collaboration

The frequency of meetings (more than once a month) with other health care professionals such as other PCPs, medical specialists, home care nurses and social workers was assessed.

#### Care activities

In this area, first, the role of PCP as first contact for different diseases was evaluated. Then, the PCPs involvement in the management of patients with different acute and chronic diagnoses was explored. For these two questions, the answer was initially assessed using a four-point scale “almost-always”, “usually”, “occasionally” and “seldom-never”. For the analysis, the two first items were secondarily grouped.

The practice of minor surgery and medical techniques was investigated. The answers, initially assessed using a four-point scale “almost-always”, “usually”, “occasionally” and “seldom-never” were presented as “seldom or never carried out” *vs* all other categories. By the same way, technical equipments of PCPs’ practices were evaluated.

#### Prevention

PCPs were asked about their involvement in domains of health prevention such as immunization of children, antenatal care and pediatric surveillance, blood pressure and cholesterol regular measures.

### Analysis

For each indicator, we calculated frequencies, or means and medians, in 1993 and 2012 and compared them using Pearson’s Chi2 test for categorical variables or t-Student test for continue quantitative variables. Nevertheless, in order to take into account the difference of the sampling method between the two surveys (stratification by canton in 2012 and by urban/rural areas in1993), the evolution between the two periods was also tested using an adjusted model on urban/rural features (logistic or linear regression according to the dependant variable studied). Analyses were performed using STATA software.

## Results

### Socio-demographic features of the samples (Table 1)

**Table 1:** PCPs‘personal characteristics in the two samples, 1993 and 2012

	1993 (*N* = 199)		2012 (*N* = 199)		1993–2012 Comparison^a^
	n	% or mean	n	% or mean	p
Sex					
Men	184	92.9	155	77.9	<0.001
Women	14	7.1	44	22.1	
					p
Age					
Mean	197	48.1	199	55.0	<0.001
Median	197	46.0	199	56.0	

^a^the results were strictly the same after adjusting on urban/rural features

The two samples included 199 primary care practitioners. Between 1993 and 2012, the proportion of women among PCPs rose from 7 % to 22 % (*p* < 0.001) and the median age increased by ten years (46 years *vs* 56 years, *p* < 0.001).

### General characteristics of PCP‘s activity (Table 2)

**Table 2:** Characteristics of PCPs’ activity in 1993 and 2012

	1993	2012	1993–2012 Comparison^a^
	% or mean (median)	% or mean (median)	p
General features			
PCP as unique activity	71.6	33.7	<0.001
Group practice	27.7	52.3	<0.001
Self-employed (*vs* salaried)	99.0	96.0	0.057
Computer equipment	78.5	100.0	<0.001
Use for making appointments	0.7	50.2	<0.001
Use for keeping records of consultations	6.0	46.7	<0.001
Use for drug prescriptions	3.3	55.3	<0.001
PC access			
Regular weekly workload as PCP (in hours)	50.2	46.6	< 0.01
	(50.0)	(48.5)	
Weekly workload (regular + after hours, in hours)	67.0	51.3	<0.001
	(64.0)	(51.0)	
Face-to-face patient contacts a day	30.9	24.0	<0.001
	(30.0)	(25.0)	
Consultation’s length (minutes)	15.1	19.6	<0.001
	(15.0)	(20.0)	
Telephone patient contacts a day	7.2	6.4	0.075
	(5.0)	(5.0)	
Home visits a week	7.7	3.2	<0.001
	(5.0)	(2.0)	
Nearest other PCP >10 km	1.5	2.0	NS
Nearest ambulatory speciality care/clinic >10 km	31.4	13.9	<0.001
Nearest hospital >10 km	31.8	25.4	NS
Regular face-to-face meeting with (>once/month)			
Other PCPs	81.9	61.2	<0.001
Hospital specialists	71.8	21.2	<0.001
Home care nurses	66.3	26.6	<0.001
Social workers	18.4	3.1	<0.001
Prevention activity involvement			
Routine antenatal care	76.8	20.7	<0.001
Immunization of children	91.8	58.3	<0.001
Paediatric surveillance < 4 years	83.5	40.1	<0.001
Blood pressure measure routinely	77.2	79.9	NS
Blood cholesterol measure routinely	13.7	36.7	<0.001

^a^the results were strictly the same after adjusting on urban/rural feature

In 1993, 28 % of PCPs used to work in group practice with other general practitioners and/or medical specialists while they are 52 % in 2012 (*p* < 0.001). In 1993, 28 % of the PCPs declared to have one or several other paid professional activities when they are 66 % in 2012 (*p* < 0.001). Teaching and practice in a residential setting are the most mentioned. The average of the physicians’ weekly workload (as PCP) decreased from 50.2 h in 1993 to 46.6 h in 2012 (*p* = 0.01). The difference is higher when after-hours are taken into consideration (67.0 in 1993 *vs* 51.3 h in 2012, *p* < 0.001). The use of computer is generalized in practices in 2012 and it is mostly used for keeping records of consultations (47 %) and making appointments (50 %).

### Access to care (Table 2)

Between 1993 and 2012, the number of daily face to face patient contacts decreased from 30 to 25 (for the medians, *p* < 0.001) but the length of consultation increased from 15 to 20 min (for the medians, *p* < 0.001). The number of telephone contacts with patients did not change but the weekly median number of home visits decreased from 5 to 2 (*p* < 0.001). The ambulatory speciality care access is better in 2012 with a diminution of those distant of more than 10 km from PCPs’ practice (31 % in 1993 *vs* 14 % in 2012, *p* < 0.01). No change is observed for the distance to the nearest hospital.

### Care continuity – collaboration (Table 2)

Face-to-face meetings with other professionals systematically decreased between 1993 and 2012. Sixty-one percent of PCPs meet other PCPs more than once a month in 2012 compared to 82 % in 1993 (*p* < 0.001) and 21 % for hospital specialist in 2012 compared to 72 % in 1993, (*p* < 0.001).

### Care activities

**Fig. 1:**
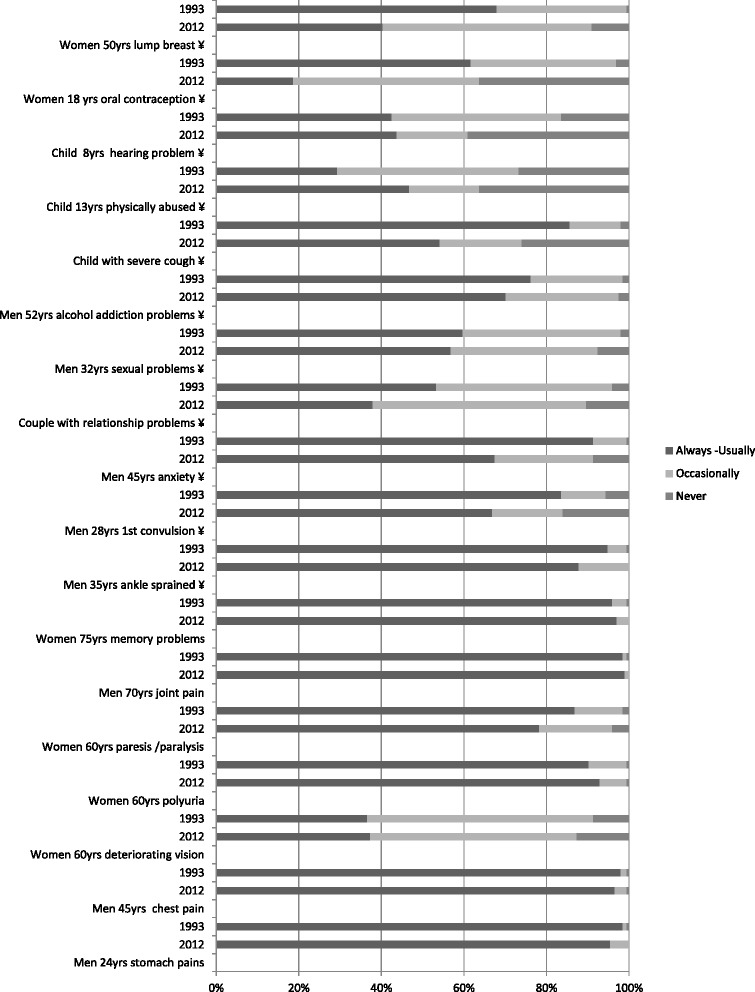
PCP as first health care provider in 1993 and 2012 (¥: *p* < 0.05)

**Fig. 2:**
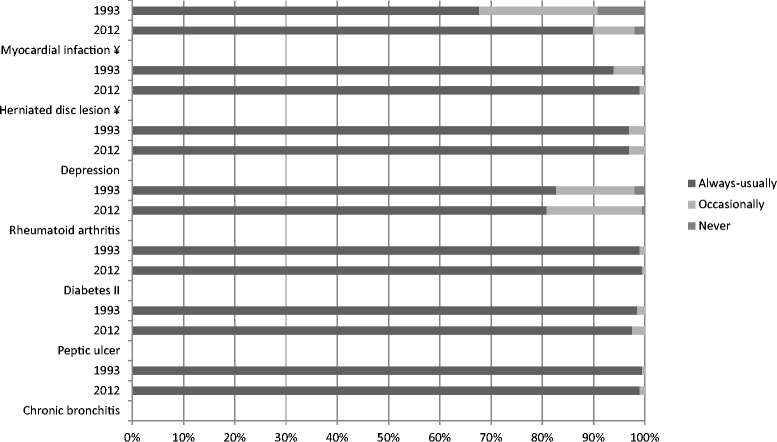
Diseases’ involvement and follow-up by PCPs in 1993 and 2012 (¥: *p* < 0.05)

Concerning the PCP‘s involvement as first health care provider, few differences are observed for problems such as common health problem of elderly people (memory problem, joint pain, deteriorating vision, polyuria…). Most of changes occurred with children‘s problem and gynecological conditions for which PCPs are generally less involved as first contact. For instance, PCPs were often involved in oral contraception’s prescription in 1993 while it is much less common in 2012 (61.7 % in 1993 *vs* 18.7 % in 2012, *p* < 0.001) (Fig. 1). The involvement of PCPs in treatment and follow-up of chronic disease little difference is observed globally between 1993 and 2012 (Fig. 2).

**Fig. 3:**
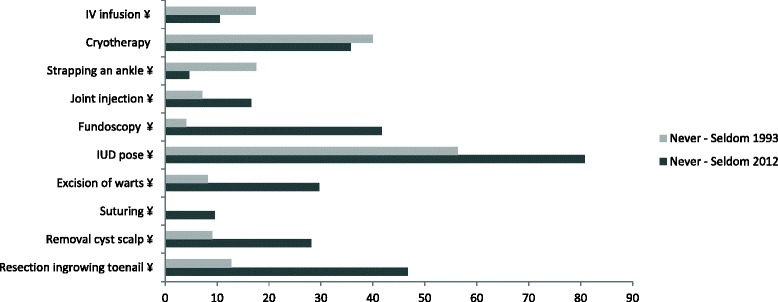
Technical activities never implemented by PCPs in 1993 and 2012 (¥: *p* < 0.05)

In 2012, the frequency of some technical activities that are “never performed” varies between 81 % (IUD pose) and 5 % (strapping an ankle). In 1993, it varied between 56 % (IUD pose) and 0 % (suturing) (Fig. 3); 8.5 % of the PCPs never performed any chirurgical acts (resection ingrowing toenail, removal cyst scalp, suturing and excision of warts) in 2012 *vs* 0 % in 1993 (Fig. 3).

### Preventive medicine (Table 2)

The activities of prevention among children (surveillance and immunization) and antenatal care are less accomplished in 2012 than in 1993 (96 % in 1993 *vs* 63 % in 2012, *p* < 0.001).

## Discussion

Our results show major changes in PCPs’ profiles and activities between 1993 and 2012. The profile of the average of the Swiss primary care physicians has changes: they are older and more feminized. Nowadays, they more often work in group practices and are more involved in other paid activities. The number of daily face-to-face contacts with patients decreased but the average duration of consultation rose. PCPs provide fewer pediatric and gynecological services but their activity remains globally unchanged in other domains. The involvement of PCPs in follow-up of chronic disease globally did not change. On the opposite, in 2012, PCPs are less involved in prevention, health education and technical activities than in 1993.

The evolution in socio-demographic features is in line with what is observed elsewhere. Feminization and aging are some features of PCPs’ demography encountered in many occidental countries [18–20]. One of the consequences of the feminization consists generally in an increase of part-time work [13, 19, 21, 22]. This issue was not directly explored in the questionnaires but could be indirectly assessed through the average number of working hours (as PCP), which decreased from around 50 h/week in 1993 to around 48 h in 2012. In 2012 as in 1993, this workload was significantly higher among men than among women. This phenomenon is amplified by the fact that more PCPs are practicing more paid activities such as teaching or in nursing home, (28 % in 1993 *vs* 66 % in 2012), independently of the gender. The resulting issue of all these changes (both demographic changes and increase of part-time working as PCP) might lead to a certain level of PCPs shortage. This will necessary leads to the need to increase the number of trained physicians as previously highlighted in a national report [23, 24]. This increase will need to be more important than just to compensate the described demographic changes as health care needs will also grow in the future, due to the fast increase of the prevalence of chronic diseases [25]. This study showed also that most practices do not have patients’ list system, which makes impossible to measure the part of the population taken in charge and its evolution. This raises the question on how to measure the shortage and its determinants: PCPs’ density, geographical heterogeneity of their distribution, reduction of the PCPs’ workload, size of the PCPs’ practice population and its consequences on primary care access for patients? The present study won’t provide a definite answer to this highly debated question. However, some elements strongly suggest that access by patients to PCP’s is more difficult than in the past, and will continue to worsen. Indeed, factors such as the increase of mean age of PCP’s, the decrease of the number of consultations per PCP (while their length increases) and the feminization are likely to lead to a certain level of shortage in some regions. Not explored in the present study is the evolution of elements related to the patients, such as the ease of access (ie. How difficult it is to get an appointment) and how the health care system responds to the perceived health care needs of the population which are also good indicators of access and indirectly related shortage.

Interestingly, despite the decrease of the weekly workload of physicians (as PCP) and a decrease of the daily number of face-to face contacts with patients, on a working day, the total contact-time with the patients didn’t change much in twenty years. Indeed, the consultations’ average duration increased over time that leads to an average daily contact-time around 7.8 h both in 2012 and 1993. This duration is important in Switzerland as compared with other European countries. In 2006, Deveugle showed that the consultation time duration was varying from 7 min in Spain and Germany to 15 min in Belgium and Switzerland [26]. This is certainly related to the Swiss payment system which is related to the consultation duration. The increase in these last twenty years is potentially related to a switch from technical activities to more psychosocial consultations that last longer.

The decrease of solo practices toward group practices is also a general trend throughout the occidental world, such as in Canada, UK or Netherlands [27]. Although the high attachment to autonomy of Swiss PC physicians, the proportion of group practices is almost twice in 2012 as compared to 1993. It is however still far from the proportion observed in Canada, where the solo practice model almost disappeared. The choice to share practices can be based on several reasons such as reducing the costs, share of knowledge, reducing the isolation in the practice; however it seems to be not always linked with a best efficiency [28].

In 2012, according to our data, all PCPs have a computer; in 1993 it concerned only 79 % of them. However, less than an half of physicians use their computer to keep records of consultation (electronic medical records, EMR). Using a computer can generate higher quality of care mainly in terms of better availability of patients’ data, better clarity of drugs prescription, better communication with other care professional [29–31]. It is to be noted that among PCP using EMR, very few are directly connected to other health care providers. Nowadays, the international debates are more centered on the definition of the best computer-based patient record system than to know if it is needed. Switzerland is definitely one of least advanced country in this domain and it becomes urgent to improve the uptake of EMR and networking of PC practices [32, 33].

The proportion of technical acts not provided by PCPs increased over the time with one exception for the ankle strapping. Both in 1993 and 2012, all these technical acts were also more implemented in rural areas than in urban ones. Some of these acts are probably now achieved in emergency departments, hospitals (IV infusion, suturing…) and clinics or by specialists’ physicians (IUD pose, excision of warts….). The hyper-specialization in medicine (increase number of gynecologists and pediatricians) can also account for the observed evolution about PCP providing less services to children and women. Concerning the follow-up and treatment of diseases, no notable change is observed except perhaps for myocardial infarctions which are more systematically followed by PCPs. This probably reflects of therapeutic progress related to this disease where more effective interventions can be provided in practices, especially to prevent complications due to myocardial infarctions [34]. Lastly, the percentage of regular (>once a month) face-to face meetings with other health professionals decreased between 1993 and 2012. This result is quite surprising but we could observe on the 2012 data that these percentages can be highly variable according to the language area in Switzerland; thus this result must be interpreted cautiously. Anyway, the lack of communication and exchange between primary care physicians and other health care specialties or structures is globally and highly reported in many developed countries [32]. A better care coordination is probably a key component to improve the health care systems’ performance in the future.

Several limitations have to be pointed out. First, the representativeness of the samples can be questioned. The two samples were drawn to be representative of Swiss PCPs. The 2012 sample was randomly drawn from a list of the Swiss PCPs stratified by canton. This sample showed a good representativeness according to sex and age, despite a low, but expected, response rate of 10 %. As data were collected more than 20 years ago, this issue is unfortunately less clear for the 1993 sample, but the representativeness in terms of sex and age was also considered as good by the investigators [10]. Moreover despite the representativeness about some major features as gender and age, other features in link with the physicians’ volunteering to participate to this survey might influence the PCPs’ practices. Lastly, the data base used in 1993 and 2012 were not strictly the same but are overlapping for the vast majority of their members. Second, even if the domains explored were the same between the two surveys, the formulation of the questions and/or the answers could be sometimes not strictly the same. For these reasons, we only kept the questions which could be directly compared without interpretation as their formulations were exactly the same. Also, the translation from the original English version sometimes differed slightly between the two surveys. This could have influenced the physicians’ answers. Despite the limitations pointed out about the evolution of the questionnaire between 1993 and 2012, it is a real asset to have Swiss data from these two European studies carried out twenty years apart. Moreover, these two databases contain only few missing data. No systematic data collection exist in Switzerland to evaluate primary care functioning, thus this study is one of the rare occasion to objectively assess the evolution of PCPs’ profiles.

## Conclusion

In the past 20 years, several changes have occurred in the Swiss primary health care system with first the introduction of a mandatory health insurance scheme (Lamal) [4] and secondly with the introduction of the homogeneous medical pricing (Tarmed) [5]. Also, the development of managed care, if observed in Switzerland in the future, might also modify the PCP’s activities [1]. This will need to be taken into consideration also in the pre-graduate curriculum of future PCP’s. The results presented here, highlighting the main changes in the PCP’s practices, are an important piece of information in the landscape of Swiss PC and are useful for medical associations, public health authorities as well as for PCP themselves. It remains however fare from being optimal in terms of accuracy of data as it relies on a small sample size. This confirms the importance to develop and implement tools allowing a more permanent and at a larger scale monitoring of PC in Switzerland.
